# A novel BRCA-1 mutation in Arab kindred from east Jerusalem with breast and ovarian cancer

**DOI:** 10.1186/1471-2407-7-14

**Published:** 2007-01-18

**Authors:** Luna Kadouri, Dani Bercovich, Arava Elimelech, Israela Lerer, Michal Sagi, Gila Glusman, Chen Shochat, Sigal Korem, Tamar Hamburger, Aviram Nissan, Nahil Abu-Halaf, Muhmud Badrriyah, Dvorah Abeliovich, Tamar Peretz

**Affiliations:** 1 Sharett Institute of Oncology, Hebrew University-Hadassah Medical Center, Jerusalem, Israel; 2 Dept of HMG & Pharmacogenetics, MIGAL-Galilee Biothechnology Center, Kiryat Shomona, Tel-Hai Academic College, Israel; 3 Department of Human Genetics Laboratories, Hebrew University-Hadassah Medical Center, Jerusalem, Israel; 4 Department of Surgery, Hebrew University-Hadassah Medical Center, Mount scopus, Jerusalem, Israel

## Abstract

**Background:**

The incidence of breast cancer (BC) in Arab women is lower compared to the incidence in the Jewish population in Israel; still, it is the most common malignancy among Arab women. There is a steep rise in breast cancer incidence in the Arab population in Israel over the last 10 years that can be attributed to life style changes. But, the younger age of BC onset in Arab women compared with that of the Jewish population is suggestive of a genetic component in BC occurrence in that population.

**Methods:**

We studied the family history of 31 women of Palestinian Arab (PA) origin affected with breast (n = 28), ovarian (n = 3) cancer. We used denaturing high performance liquid chromatography (DHPLC) to screen for mutations of BRCA1/2 in 4 women with a personal and family history highly suggestive of genetic predisposition.

**Results:**

A novel BRCA1 mutation, E1373X in exon 12, was found in a patient affected with ovarian cancer. Four of her family members, 3 BC patients and a healthy individual were consequently also found to carry this mutation. Of the other 27 patients, which were screened for this specific mutation none was found to carry it.

**Conclusion:**

We found a novel BRCA1 mutation in a family of PA origin with a history highly compatible with BRCA1 phenotype. This mutation was not found in additional 30 PA women affected with BC or OC. Therefore full BRCA1/2 screening should be offered to patients with characteristic family history. The significance of the novel BRCA1 mutation we identified should be studied in larger population. However, it is likely that the E1373X mutation is not a founder frequent mutation in the PA population.

## Background

Breast cancer (BC) is a leading cause of cancer morbidity and mortality among Israeli women regardless of their ethnic origin. Although the incidence of BC is lower in the Arab (PA) compared with the Jewish population in Israel, it is continuously increasing during the last decades [1]. In our previous study conducted on a series of 312 women, we found significantly younger age at onset and advanced stage at presentation among Palestinian Arab (PA) women compared with Israeli Jews [2]. Similar results were reported for other Arab countries in the Middle East [3,4]. These characteristics of BC in the Arab population raise the need for development of screening programs for early detection and the defining of high risk individuals in that population.

Young age at diagnosis in a cohort of BC patients may be associated with a genetic predisposition such as the presence of a BRCA1 or BRCA2 mutations in Ashkenazi women [5]. A recent report of the Israel Center of Disease Control showed a higher risk of BC in Arab women with family history of BC in Israel [6].

The role of BRCA1/2 mutations in the Arab population was understudied; therefore the genetic component and the contribution of the BRCA1/2 genes to BC or ovarian cancer (OC) in that population are unknown. Only 5–10% of newly diagnosed BC patients and about 5% of OC cases are attributable to high penetrant breast cancer predisposing genes [7]. BRCA1 and BRCA2 can be identified in about half of the breast cancer patients with positive family history [7]. Higher frequency of BRCA1 and BRCA2 germ-line mutations were found in Ashkenazi Jewish breast and ovarian cancer patients regardless of family history [5]. Up to now, only two BRCA mutations, a novel BRCA2 mutation (2482delGACT) and a BRCA1 mutation (Arg841Trp) were reported in Arab BC patients from Saudi-Arabia [8].

The present study was conducted on a population of 31 PA women who were treated in our institution for BC or OC and referred to the onco-genetic clinic. The majority of them were diagnosed below of age 50 years and/or presented with a family history of BC. Full sequencing was done in 4 of them with a personal and family history suggestive of a genetic predisposition. After identifying a BRCA1 mutation, the rest of the group was screened for that mutation.

## Methods

### Study population

Thirty-one women of Palestinian origin affected with breast (n = 28) or ovarian (n = 3) cancer were referred for genetic counseling in the Cancer Genetic Clinic in Hadassah medical center during 1995–2002. All participants signed an informed consent approved by the institutional ethics committee. Clinical characteristics of study population are presented in table 1. Of 28 women affected with BC, 12 were diagnosed before age 40, 7 at age 41–50 and 9 above age 50 years. Family history of BC was recorded in 5 of them and additional 3 women reported a history of 3 or more cases of BC in the family. Among 3 women affected with OC, 2 had family history of BC. A history of other cancers (pancreas, prostate, leukemia, lymphoma, lung, and others) was reported in 13 families (in 4 of them, it was in addition to BC history).

**Figure 1:**
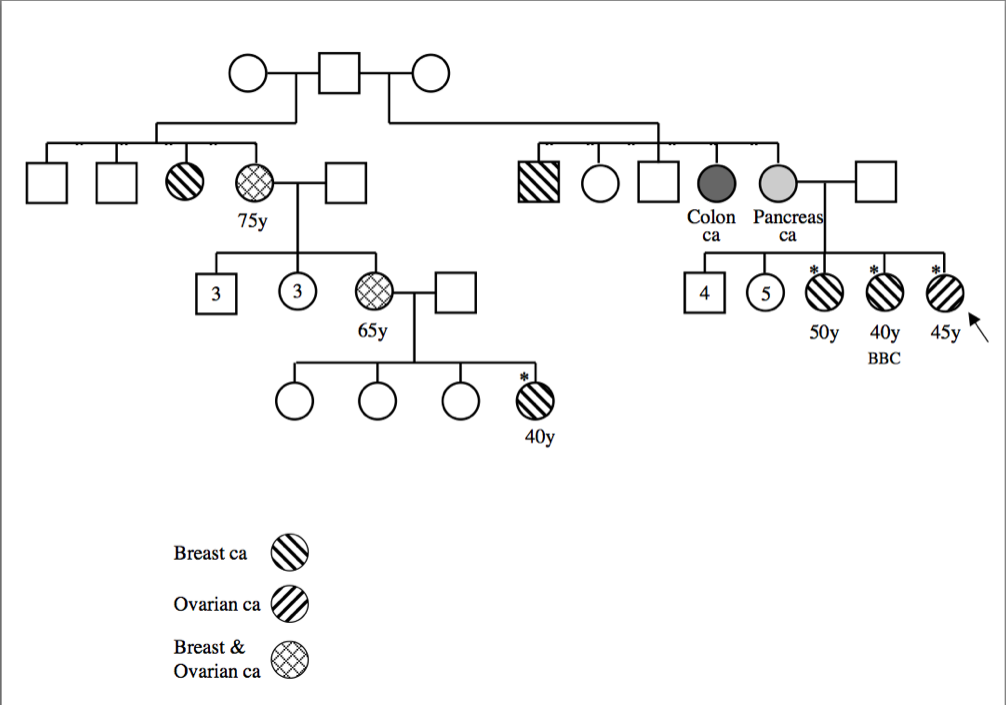
Family tree of a patient of Palestinian Arab origin (DNA no. 8) found to carry the E1373X-BRCA1 mutation.

Full screening and sequencing of BRCA1 and BRCA2 genes was performed on DNA from four women with a family history highly suggestive of genetic predisposition. Of the 3 OC patients, one individual, who was diagnosed at age 45 and had 3 sisters affected with BC (one of them had bilateral BC, see family tree in figure 1), was analyzed. In addition, of 12 young onset BC patients, 3 women with the most suggestive combination of age at onset and family history were chosen for screening. Therefore, 3 patients affected at ages: 24 (with 2 first degree relatives affected with BC); 31 (with an aunt affected with BC and other relatives affected with lung and pancreatic cancer) and 38 (with 3 sisters affected with BC) were analyzed.

### Genotype analysis

Genomic DNA was extracted according to standard protocols for mutation analysis. DNA samples were used as a template for PCR and DHPLC screening analysis.

### Establishing DHPLC conditions

Denaturing high-pressure liquid chromatography (DHPLC, Transgenomic, Omaha, NE, [10]) and direct sequencing were used to screen stored leukocyte genomic DNA for mutations within the full BRCA1 & 2 genes sequences. Scanning for mutations or polymorphism by DHPLC involves subjecting PCR products (unpurified) to ion-pair reverse-phase particles. Under conditions of partial heat denaturation within a linear acetonitrile gradient, heteroduplexes that form in PCR samples having internal sequence variation display reduced column retention time relative to their homoduplex counterparts. The elution profiles for such samples are distinct from those having a homozygous sequence, making the identification of samples harboring polymorphisms or mutations a straightforward procedure. Both upstream and downstream DNA sequencing were performed to characterize the exact sequence variation of different chromatograms detected by the screening with the DHPLC [11]. The DNA screening was performed using a WAVE apparatus from Transgenomic Inc. (Omaha, NE). The PCR products were denatured at 95°C for 5 min and cooled to 65°C down a temperature ramp of 1°C/min. The samples are kept at 4°C until 5 μl are applied to a preheated C18 reversed phase column based on non-porous poly (styrene-divinyl-benzene) particles (DNA-Sep Cartridge, CAT no. 450181; all DHPLC catalog numbers are Transgenomics Inc.). DNA was eluted within a linear acetonitrile gradient consisting of buffer A (0.1 M triethylammonium acetate (TEAA) – CAT no. SP5890)/buffer B (0.1 M TEAA, 25% acetonitrile – CAT no. 700001). The temperature at which heteroduplex detection occurred was deduced from the Transgenomic software (Wavemaker 4.1) and The Stanford DHPLC melting program [12], which analyzes the melting profile of the specific DNA fragment. Each exon was screened for the BRCA1 (31 fragments) and BRCA2 (40 fragments). PCR products with alter chromatograms were sequenced using ABI 310 sequencer.

### The assay for the mutation E1373X in the BRCA1 gene

**Figure 2:**
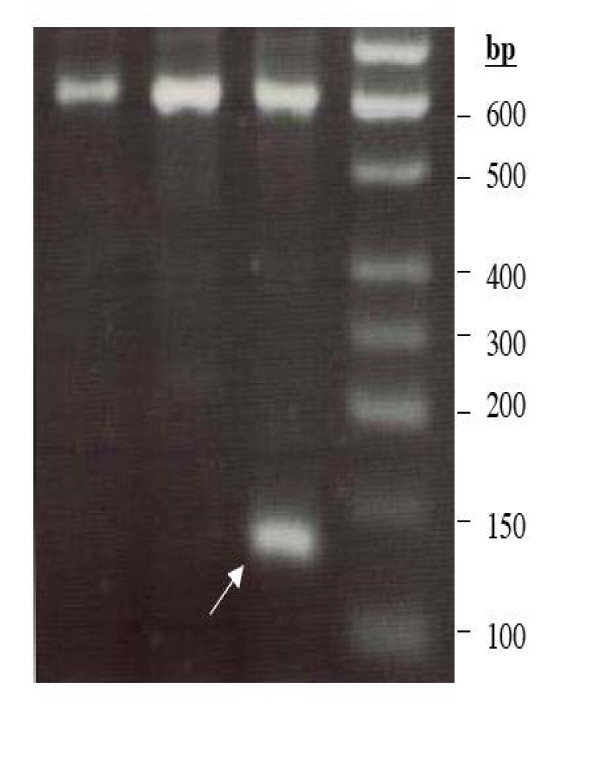
A gel picture pf PCR product of E1373X mutation analysis. The E1373X mutation generated a PCR product of 120 bp (marked by an arrow) and the normal allele does not yield any product. A control PCR generated a 600 bp product from both the mutant and normal alleles.

The mutation was tested in a multiplex PCR, using allele specific oligonucleotide primers, 12-F: 5'-CTG CTG CCA ATG AGA AGA AA-3'; 1373X R: 5'-CTT CAG AGA CGC TTG TTT CAC TCG GA-3'. The E1373X mutation generated a PCR product of 120bp, the normal allele does not yield any PCR product. A control PCR generated a 600bp product from both the mutant and normal alleles. All mutation analyses included positive and negative controls for the mutation, confirmed by sequencing (fig. 2).

## Results

Table 2 presents mutations and polymorphisms, which were found among the 4 patients (DNA numbers 8–11). A total of 16 alterations were found In the BRCA1 gene: 1- nonsense mutation, 6 missense mutations (5 of them are polymorphisms and one, a mutation designated M1652I, of unknown importance according to the BIC database [13]), 3 IVS, 5 silent and 1 5UTR.

The BRCA1 nonsense mutation, designated E1373X is in exon 12. It was found in a patient diagnosed with ovarian cancer at age 45. The family tree is presented in figure 1. Following the detection of the mutation additional 4 family members were tested for this mutation, 3 were found to be carriers of this mutation. Two women affected with BC (one at age 40 and the other at age 50, one of them had bilateral BC) and one was a healthy individual aged 32. Screening of the other 30 patients for the E1373X-BRCA1 mutation revealed no other carrier of this mutation.

The missense mutation in exon 16, M1652I, with unknown importance according to the BIC database [13] was found in a women diagnosed at age 31 with a family history of an aunt with BC and a mother affected with lung cancer. The mutation could not be screened in additional family members to further elucidate the role of this mutation.

No disease causing mutation was found in the BRCA2 gene. Seven DNA alterations were found: 2 IVS, 3 silent mutations, 1 5UTR and 1 missense mutation (categorized in the BIC database [13] as polymorphism).

## Discussion

In the present study, which follows a previous clinical and pathological analysis of a cohort of BC patients of PA origin [2], we focus on identifying BRCA1/2 mutations among BC/OC PA patients with suggestive family history. In 4 patients screened for BRCA1/2 mutations, we have found one nonsense BRCA1 mutation, a G to T base change located at base 4236, which translates to a stop codon at codon 1373. The family history is a typical for BRCA1 carrier family, with women affected with BC and OC, young age at onset of BC and a history of bilateral BC [5]. A history of male BC, which was recorded in the family, is a typical feature of BRCA2, although BRCA1 carrier status was associated with male BC as well [14]. The other 3 women, in whom no mutation was found were diagnosed with BC at young age and had a family history of BC.

Recently we reported [2] a clinico-pathological analysis of three ethnic groups in a cohort of 312 women affected with BC. Survival, age and stage at diagnosis were compared between Ashkenazi Jews (AJ), Sephardic Jews (SJ) and Palestinian Arabs (PA) in Jerusalem. The mean age at diagnosis was 51.5 years for the PA group and 55.9 years for the AJ group (p < 0.03). Eleven percent of the PA patients were under the age of 35 as compared to only 5% of the two Jewish (Ashkenazi and non-Ashkenazi) study groups. A study from the Kingdom of Saudi Arabia [3] reported an overall low incidence of breast cancer in the population studied. However, 78% of the women were diagnosed before the age of 50 years and 79% were premenopausal at diagnosis. Another study reported by the same group [4] showed young age and advanced stage at presentation of breast cancer patients with 64% being younger than 50 years at the time of diagnosis and 62% being premenopausal. These data suggests role for a genetic predisposition in addition to environmental and hormonal risk factors in BC development in this population. The young onset of BC among PA women is also attributed to the revolution in life style factors such as the increased utilization of hormones in form of contraceptives or fertility treatments that leads to steep rise in the incidence of BC in that population. In that case, the increase in BC incidence is first manifested in the younger age group at risk and later as the cohort gets older it will gain its accurate distribution by age. Still, genetic predisposition may be modified by the rapid change in life style and hormonal factors in this population. Therefore, this understudied population can provide important data regarding gene-environment interactions in well designed studies. Genetic studies may also aid in population education and compliance with early detection screening programs.

In addition to full BRCA1/2 sequencing of 4 DNA from the above mentioned patients we also screened 27 DNA samples from affected PA individuals for this BRCA1 mutation (E1373X). We did not find additional carrier in this group. Only one previous report of a BRCA2 mutation (2482delGACT) found in a BC patient from PA descent was published [8]. Zlotogora et al [15,16] studied and reviewed the inheritance of genetic disorders in the PA population. The Arab population is genetically heterogeneous; therefore the distribution of genetic disorders in this population is not uniform. Most of the PA population in Israel and in the Palestinian Authority lives in villages/tribes that were founded by few individuals less than 10 generations ago and often includes less than 10,000 inhabitants. Consanguineous marriages are frequent in this population, therefore, each of the villages may be considered as a small isolated community [16]. Indeed, there are diseases that are prevalent in some villages and rare or absent in others, while diseases that are relatively frequent among Muslim Arabs are more homogenously prevalent in the PA population in Israel as well [16]. Therefore, our study may result in various private mutations responsible for morbidity in relatively small communities.

## Conclusion

We attempted to delineate the genetic component of BC/OC among the PA population. However, only full sequencing of the BRCA1/2 genes and study of the particular BRCA1 mutation that we identified in a larger population may provide complete picture regarding the role of BRCA1/2 mutations in the studied population. Based on our study full BRCA1/2 screening should be offered to families with a history highly suggestive of genetic predisposition. It is likely that the E1373X mutation is not a founder frequent mutation in the PA population.

## Competing interests

The author(s) declare that they have no competing interests.

## Authors' contributions

LK participated in design of study and writing the paper

DB participated in design of study, designed DHLPC assay for BRCA1/2 screening

AE carried out DHLPC assay and sequencing for BRCA1/2 screening

IL designed assay for mutation analyses

GG carried out mutation analyses

MS established with TP the onco-genetic clinic, participated in design of study and drafting the paper

CS carried out DHLPC assay and sequencing for BRCA1/2 screening

SK carried out DHLPC assay and sequencing for BRCA1/2 screening

TH collected clinical data and drafted the paper

AN participated in design of study, writing and drafting the paper

NA participated in design of study and collection of DNA samples

MB participated in design of study and collection of DNA samples

DA participated in design of study and drafting the paper

TP established with MS the onco-genetic clinic, participated in design of study and drafting the paper

All authors read and approved the final manuscript

## Pre-publication history

The pre-publication history for this paper can be accessed here:



## Figures and Tables

**Table 1:** clinical characteristics of study population: diagnosis, age at onset and family history.

	Breast Cancer (n = 28)			Ovarian Cancer (n = 3)		
Age at onset (years)	<40 (n = 12)	41–50 (n = 7)	>50 (n = 9)	45^3^	56	60
Family History:						
BC^1^(n)	3	2	3	yes	yes	no
Other Cancers^2^(n)	5	4	4	yes	no	yes

^1^In 3 patients a history of 3 or more cases of BC was reported.

^2^Type of other cancer diagnosis in the families included: pancreas, prostate, leukemia, lymphoma, lung and others.

^3^Carrier of the E1373X BRCA1 mutation.

**Table 2:** BRCA1/2 mutations and polymorphisms in 4 Palestinian Arab women affected with breast (DNA no. 9–11) and/or ovarian cancer (DNA no. 8) identified using DHLPC and sequencing.

**NO. OF DNA**	**Exon, Gene**	**NT**	**Codon**	**Base Cange**	**Mutation Type**	**Designation**	**AA Cange**
**8**	**12 BRCA1**	**4236**	**1373**	**G to T**	**Nonsense**	**E1373X**	**E to STOP**
**11**	**16 BRCA1**	**5075**	**1652**	**G to A**	**Missense-UV**	**M1652I**	**M to I**
9	1BRCA1	-233 from 5UTR		g to a	IVS	-233 from 5UTR g/a	
8	IVS6 BRCA1	IVS6 +62		t to c	IVS	IVS6 -62 t/c	IVS
9,10,11	IVS7 BRCA1	IVS7 -65		Del a		IVS7 -65 del a	IVS
9	11.3 BRCA1	1868	583	A toG	K to K	K583K	Silent
11	11.3 BRCA1	1955	612	G to A	R to R	R612R	Silent
9,10,11	11.4 BRCA1	2196	693	G to A	D to N	D693N	Missense -P
9,10,11	11.4 BRCA1	3001	694	C to T	S to S	S694S	Silent
9,10,11	11.5 BRCA1	2430	771	T to C	L to L	L771L	Silent
9,10,11	11.5 BRCA1	2731	871	C to T	P to L	P871L	Missense-P
8,9,10,11	11.7 BRCA1	3232	1038	A to G	E to G	E1038G	Missense-P
8,9	11.8 BRCA1	3667	1183	A to G	K to R	K1183R	Missense-P
9,10,11	13 BRCA1	4427	1436	T to C	S to S	S1436S	Silent
8,9,10,11	16 BRCA1	4956	1613	A to G	S to G	S1613G	Missense-P
8,9,10,11	IVS17 BRCA1	IVS17+66		g to a		IVS17 +66 g/a	IVS
8,9,11	2BRCA2	202		g to a		5UTR -27 g/a	
11	IVS2 BRCA2	IVS2 -7		t to a		IVS2 -7 t/a	IVS
11	IVS8 BRCA2	IVS8 +56		c to t		IVS8 +56 c/t	IVS
8,10	10.2BRCA2	1342	372	A to C	H to N	H372N	Missense-P
8,9,10,11	11.4BRCA2	3624	1132	A to G	K to K	K1132K	Silent
9	11.5BRCA2	4035	1269	T to C	V to V	V1269V	Silent
8,9,10,11	14BRCA2	7470	2414	A to G	S to S	S2414S	Silent

Missense-UV – according to BIC database [13]

Missense-P – polymorphism according to BIC database.

IVS – Intronic sequences variation

Silent – no amino acid changes
